# Mock-Ups in Plastic Conservation Research: Processing and Aging of 3D Celluloid Specimens Simulating Historical Objects

**DOI:** 10.3390/polym15040852

**Published:** 2023-02-08

**Authors:** Christina Elsässer, Veronika Mayr, Peter Montag, Eva Mariasole Angelin, Harald Hilbig, Christian Ulrich Grosse, Marisa Pamplona

**Affiliations:** 1 Conservation Science Department, Deutsches Museum, Museumsinsel 1, 80538 Munich, Germany; 2 Chair of Non-destructive Testing, Technical University of Munich, Franz-Langinger-Straße 10, 81245 Munich, Germany; 3 PSS, Polymer Standards Service GmbH, In der Dalheimer Wiese 5, 55120 Mainz, Germany; 4 Professorship of Mineral Construction Materials, Technical University of Munich, Franz-Langinger-Straße 10, 81245 Munich, Germany

**Keywords:** celluloid, camphor, cellulose nitrate, three-dimensional mock-ups, artificial aging, degradation, ion chromatography, gel permeation chromatography, ATR-FTIR spectroscopy

## Abstract

The preparation of mock-ups in heritage science studies represents a valid alternative for investigation purposes, avoiding extensive sampling of cultural heritage objects. This work presents for the first time the successful preparation of three dimensional (3D) mock-ups made of celluloid, considering a combination of historical industrial production strategies and small-scale lab facilities. Prefabricated transparent celluloid sheets were acquired and then shaped through compression molding for creating mock-ups with 3D geometries. These reflected common and representative shapes encountered in the collection of the Deutsches Museum. Visual inspection of the mock-ups allowed determining the best compression molding conditions. Attenuated total reflection Fourier-transform infrared spectroscopy (ATR-FTIR) confirmed the absence of molecular heterogeneity due to the processing method. Artificial aging of the mock-ups was conducted to reach degradation states comparable with naturally aged objects. ATR-FTIR investigation offered first insights into the induced artificial degradation. Ion chromatography (IC) and gel permeation chromatography (GPC) analyses allowed to assess the extent of the artificial aging of the celluloid mock-ups and confirmed the occurrence of loss of camphor, denitration, and main chain polymer scission, the latter being the predominant decay path. The comparison with historical objects highlighted that the mock-ups are representative of moderately aged artifacts. As such, this study paves the way for implementing moderately aged celluloid 3D mock-ups in heritage science research, enabling in-depth testing for the scope of conservation.

## 1. Introduction

Celluloid counts as one of the milestones in the history of polymers, since it marked the starting point for the commercial use of plastics at the end of the 19th century [1] and the beginning of our plastic age [2]. Celluloid is a semisynthetic plastic made of cellulose nitrate (CN) plasticized with terpenoid camphor. Thanks to its versatile properties, celluloid was used in many applications to produce cinematographic films, photographs, textile fibers, and molded goods, but also renowned constructivists of the 20th century appreciated this material for their artworks. Therefore, three-dimensional (3D) artifacts made of celluloid are present in various collections, including design, science, technology, and modern and contemporary art. Because of its inherent instability, celluloid is considered one of the malignant plastics and is challenging to preserve in collections. The comprehensive understanding of its complex deterioration and the exploration of conservation treatments for its efficient safeguarding are still research subjects in heritage science.

As artifacts are testimonies of our past, with a diversity of values including historical, artistic, scientific, and social significance, they should be treated with great respect. In heritage science studies, destructive testing methods yield more detailed information than nondestructive techniques and can be necessary for a thorough characterization. However, those destructive approaches should limit the collection of samples in amount and location. To minimize damage due to sampling, mock-ups can be considered a valid alternative. Mock-ups are usually made of a defined material composition and with a specific method, with the goal of simulating the materiality of cultural heritage objects as closely as possible. Because their sampling does not present any ethical limitation, mock-ups allow in-depth studies. In this framework, mock-ups ensure reproducible and reliable testing material, leading to exhaustive and statistically relevant data, which can be implemented to understand the objects’ properties and behavior, as well as to help to evaluate the effects of active and passive conservation treatments.

Frequently, mock-ups are prepared in conservation research for imitating paint layers on different substrates [3,4]. Until now, mock-ups in plastic conservation research comprise thermoplastic polymers and elastomers for aging, mechanical, and conservation treatment studies. They are normally prepared (i) by using mass-produced granulates and industrial processing machines for their shaping [5] and (ii) by employing prefabricated plastic sheets or plates without further processing [5,6,7,8,9,10], the latter approach being the most common. However, creating representative plastic mock-ups at the lab scale is rather challenging, as their production is not always straightforward or comparable to industrial processing methods. Even though industrial manufacturing methods are available today to conservation scientists, those methods might differ from the ones used in the past. Plastic formulation (including additives, quality, and quantity), kind of processing (e.g., injection molding, casting, calendaring, extrusion), and parameters (e.g., temperature, pressure, timings of different process phases) can greatly vary. To the best of the authors’ knowledge, only the Getty Conservation Institute uses a “miniaturized plastic plant” to simulate an industrial production scenario in the laboratory to create cellulose acetate (CA) mock-ups [11]. The selection of the raw materials and their processing methods should be carefully considered in the creation of plastic mock-ups, as they will strongly influence the properties and long-term behavior of the produced specimens. This selection, virtually as close as possible to the one used in the past to produce historical objects, will determine the suitability of the mock-ups.

Regarding celluloid research, mock-ups would advance conservation professionals’ knowledge without the need for an extensive sampling of historical objects. Published studies involve the preparation of celluloid shaped as 2D films and 3 mm-thick disks by either synthesizing the cellulose nitrate, by mixing cotton linters with nitric and sulfuric acid and adding camphor [12], or by solving industrially manufactured cellulose nitrate membranes and combining them with camphor [13,14]. The resulting celluloid was cast or pressed into the desired form using simple lab facilities [12,13,14] without using industrial processing machines to formulate and shape. However, their production is not yet established in what concerns mock-ups simulating 3D celluloid objects.

This work aimed to prepare 3D mock-ups made of celluloid considering the combination of historical industrial production strategies and small-scale lab facilities. After acquiring prefabricated celluloid sheets and processing them to mock-ups, these were artificially aged to reach a moderate condition intending to resemble naturally aged objects in museum collections. The investigation comprised the assessment of macroscopic defects and possible molecular changes of the mock-ups due to shaping and processing, and their degradation extent due to artificial aging. To verify the representativeness of the mock-ups before their implementation in a research project [15] (aiming to evaluate different storage temperatures for the preservation of 3D celluloid artifacts), the artificially aged mock-ups were compared with naturally degraded celluloid historical objects. 

Attenuated total reflection Fourier-transform infrared spectroscopy (ATR-FTIR) was used as a preliminary investigation tool to investigate the variation of selected probe bands related to the fingerprint of celluloid after processing and before artificial aging. Following the strategy proposed by [16], the extent of degradation was assessed in detail by ion chromatography (IC) and gel permeation chromatography (GPC). These destructive techniques allow the investigation of the leading chemical and physical alteration processes of celluloid, namely, the breakdown of the polymer, denitration with the emission of nitrous gases, and the loss of plasticizers, particularly camphor [16,17,18,19,20,21,22,23,24].

## 2. Materials and Methods

### 2.1. Mock-Ups: Reflection on Past Industrial Celluloid Processing and Selection of Starting Materials and Geometries

In the past, companies in charge of CN synthesis and celluloid production were usually different from those shaping the 3D goods (e.g., combs, toys, eyeglass frames, instrumental keys, technical instrument dials, and jewelry). Companies would buy prefabricated celluloid sheets in the thickness of the final product to further shape them, either by machining or heat-involved forming techniques [25,26,27,28].

Due to contemporary safety, environmental, and health regulations, celluloid is hardly produced by the plastic industry worldwide. Therefore, only very limited prefabricated celluloid plates are available nowadays, with specific formulations and thicknesses. Taking into account this constraint, transparent colorless celluloid sheets (Incudo Clear Transparent Celluloid Sheet RF0425, 430 mm × 290 mm × 0.5 mm) from Rothko and Frost™, available in Europe, were purchased. In this study, the work by the authors corresponds to the processing of prefabricated sheets, following historical production strategies as close as possible. Besides the main chemical constituents, no other information was available from the producer Rothko and Frost. Transparent celluloid was considered in this investigation because it represents the basic formulation and is acknowledged to be less stable than opaque celluloid in museum collections [19,27]. Three mock-up geometries were produced based on the celluloid artifacts surveyed at the Deutsches Museum (DM): sheets representing industrial plastic swatches, tines recreating the geometry found in combs, and cylinders with a metal core referring to the geometry found in eyeglasses [29]. The dimensions for each mock-up geometry are described in Table 1, and 100 mock-ups were made per geometry. XRF analyses confirmed a predominant usage of nickel silver (an alloy made from copper, nickel, and zinc) in eyeglasses from the DM; therefore, nickel silver rods 2 mm in diameter (Gemmel Metalle) were used to process the cylinder mock-ups. Since the mock-ups were aimed at testing storage conditions in a future research project, depths between 0.5 and 10 mm were considered, as these would offer the chance to investigate the influence of temperature on the stability of 3D celluloid with different thicknesses [15].

### 2.2. Mock-Ups: Instrumentation for the Shaping Process

To gain the geometries shown in Table 1, the sheets were cut from the prefabricated sheets into the desired dimensions and were ready for usage without further manipulation. For the production of tines and cylinders, the prefabricated sheets were cut and then shaped under a heatable slab press (PCS II, COLLIN) for compression molding. Compression molding with heat and pressure was employed because it was one of the past’s standard methods for producing combs and spectacle arms [25,26,28]. The molds were designed with CAD and manufactured in aluminum (tines) and steel (cylinders).

### 2.3. Selection of Compression Molding Parameters for Processing Tines and Cylinders

**Figure 1:**
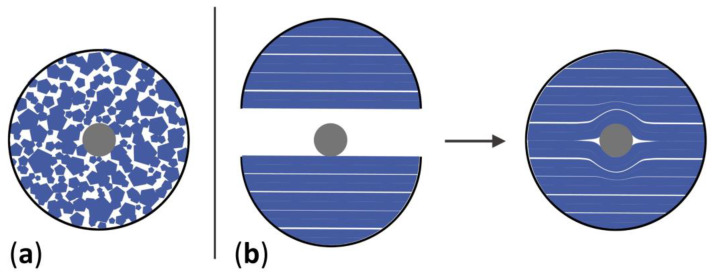
Diagram illustrating how the granules (**a**) and sheets (**b**) were piled to fill the cylinder mold.

Because the prefabricated 0.5 mm celluloid sheet did not match the thickness required for the tines and cylinders, the following adjustments were made. The prefabricated sheets were cut in geometries following the contour of tines or cylinders, or cut into tiny pieces to simulate industrial granulates, and then layered or pilled in the mold to produce thick 3D shapes (Figure 1). The precut geometries or granulates were softened with solvent to ensure better cohesion between layers. Acetone (≥ 99.5% for synthesis, Carl Roth) was chosen as a solvent instead of ethanol because it evaporates faster and dissolves celluloid more effectively [21,26,30].

As the processing greatly influenced the final-product properties, the following parameters were considered:

- raw material (granules and sheets);
- time between softening and pressing (4 h, 1 day, 2 days, 6 days);
- set temperature of the slab press (90, 100, 120 °C);
- preheating time (for tines: 3, 5 and 7 min; for cylinders: 10, 15, 20 min);
- pressure (0 and 25 bar);
- pressing time (5, 8, 10 min);
- cooling method (after pressing, the mold was either opened directly (no cooling), cooled by the cooling function of the press (slow cooling) or cooled by putting it in a water (fast cooling)).

These were tested in different combinations to assess the best compression molding procedure. Only one parameter was changed per production test, and the raw material, preheating time, pressure, and cooling method were the first to be defined, being the other parameters tested in every possible combination. The set temperature of the press and pressure were chosen following historical sources [25,26,28]. The preheating time, pressing time, and cooling method were derived from DIN EN ISO 293 “Plastics—Compression moulding of test specimens of thermoplastic materials” [31].

The quality of the produced mock-ups was assessed visually after production. To select the best combination of processing conditions, both time efficiency and the occurrence of minimal or no defects (e.g., blowholes, bubbles, deformation by demolding, discoloration, incomplete filling of the mold, irregularities on the surface, cavities and sunken areas at the surface, warpage, and shrinkage, deviation of the metal rod from the cylinders’ centers) were taken into consideration.

### 2.4. Artificial Aging

Artificial aging can help to speed up deterioration processes for studying their reactions in the laboratory [32]. In this study thermohygrometric artificial aging was applied to accelerate the deterioration processes in the newly processed mock-ups following the strategies presented in Table 2. The artificial aging aimed at inducing a moderately degraded condition. Therefore, the mock-ups were monitored by visual assessment and mass loss. The duration of the artificial aging was determined when mock-ups were visibly in good condition but off-gassing (acid detection).

A fan-assisted dynamic climate chamber (MKF 115, Binder, Tuttlingen, Germany) was used to age sheets, tines, and cylinders. Unaged mock-ups for each geometry were kept in the dark at room temperature as control.

Sheets. Eighty sheets were artificially aged at 70 °C and 75% relative humidity (RH), following Quye et al. [12] and Steward [36,37]. A hole was drilled in one of the corners to allow their hanging on a glass rod, permitting air circulation during aging in the chamber. Every working day, eight sheets were weighed, and during this measuring time (around 20 min), the door of the climate chamber was left open to allow a complete exchange of air.

Tines. The conditions of the artificial aging were 60 °C and 75% RH. Forty tines were placed in petri dishes without being in contact with each other. Following the same strategy as for sheets, eight tines were weighed following the same procedure. The tines were weekly turned upside down on the petri dishes to homogeneously expose their surfaces.

Cylinders. The cylinders were aged together with the tines at 60 °C and 75% RH sharing the same environment. As for the tines, 40 cylinders were placed in petri dishes without touching each other and weekly turned during the aging. The cylinders were weighted as for sheets and tines.

More information about the rationale behind lowering the temperature in the aging of tines and cylinders is reported in Section 3.3 and the Supplementary Materials (Figure S1 and related paragraph).

### 2.5. Characterization of Mock-Ups

#### 2.5.1. Weight Measurements

Weight measurements were performed with a Practum 224-1x (Sartorius) balance at room temperature in the laboratory (precision ± 0.1 mg). Before, during, and after the artificial aging, samples of each mock-up geometry were measured, and the percentage mass losses during time were calculated.

#### 2.5.2. Off-Gassing Control with A-D Strips

A-D strips (Danchek AD Strips), an acid–base indicator made of paper strips dye-coated with bromocresol green indicator developed by the Image Permanence Institute (IPI), were used to determine the presence of acids associated with off-gassing from the mock-ups after artificial aging. The indicator will change from blue, via green, to finally yellow in the presence of acids depending on the pH. Even though such A-D strips were designed for acetate films [42], they are also applied to detect celluloids’ nitric acid emission [43,44]. Four samples of each mock-up geometry were bagged individually in a 7 × 10 cm PE-LD zipper bag (WeltiesSmartTools Premium) with an A-D strip close but not in contact with the samples. The PE-LD bags were then stored in the dark in a lab environment, and the color of the A-D strips was visually evaluated after 24 h of exposure.

#### 2.5.3. Attenuated Total Reflection Fourier-Transform Infrared Spectroscopy (ATR-FTIR)

ATR-FTIR analysis was applied to (a) assess if the processing methods of the mock-ups in this study induced unwanted heterogeneity at molecular level of the tines and cylinders, and (b) estimate their degradation caused by artificial aging.

For both aims, the mock-ups were characterized with a Bruker Alpha ATR-FTIR spectrometer equipped with a diamond crystal. The collected spectral region was 4000–400 cm^−1^ using 64 scans with a spectral resolution of 4 cm^−1^. Opus 8.1 software (Bruker Optics) was used to acquire and elaborate the spectra.

In order to check the possible occurrence of heterogeneities in the tines and cylinders due to their processing, the standard deviation (SD) of the absorbance intensities of a selection of ATR-FTIR bands was calculated following Ballany [45] and Shashoua and Johansen [46]. The unaged sheets, neither manipulated by the shaping process nor the aging, were used to measure the instrumental and sampling errors that would set the SD ranges. The instrumental errors were determined by running ten spectra at the same spot (experiment A). Sampling errors were considered variations in the spectra resulting from ten different positions on one sheet (experiment B) and ten different sheets (experiment C) (Table 3). All acquired spectra were normalized by using the peak of C-O-C in the backbone of anhydroglucose units at 1051 cm^−1^ as reference (A/A1051cm^−1^). This peak is supposed to be less affected during degradation and is considered unchanged in the early stages of degradation [12,36,47,48,49]. After normalization, the SDs of the absorbance intensities at 1730 (C=O), 1634 (NO_2_), 1273 (NO_2_) and 826 cm^−1^ (NO) were calculated. The minimum and maximum values of SDs achieved from experiments A to C gave the ranges of allowable variations for each peak. Heterogeneity due to the processing would be evident if the SD values of each absorbance intensity for the tines and cylinder were higher than the maximum allowable SD value calculated from the sheets.

Table 4 depicts the measuring and sampling strategy used to assess the possible heterogeneity after processing and the degradation after artificial aging. While measurements of the sheets could be performed directly on five locations at the surface, the irregular and rounded shapes of the tines and cylinders inhibited comparable band intensities and quality of the IR spectra due to unequal contact with the diamond crystal. Therefore, tines and cylinders were sampled with a surgical scalpel at six sampling spots under the stereomicroscope, and the sampled chips were subjected to ATR-FTIR analysis straight after the sampling. Each chip was handled under magnification lenses to ensure the measurement of its outer surface. One ATR-FTIR measurement was performed for each location, and four samples per mock-up geometry were considered to guarantee representativeness. All acquired spectra were normalized (A/A1051cm^−1^). Absorbance intensities at 1634, 1273 and 826 cm^−1^ associated with nitro groups and at 1730 cm^−1^ originating from carbonyl stretching were selected as probe bands to estimate the degradation due to artificial aging.

#### 2.5.4. Sampling Strategy for the Quantitative Analysis of the Chromatographic Methods (IC and GPC)

**Figure 2:**
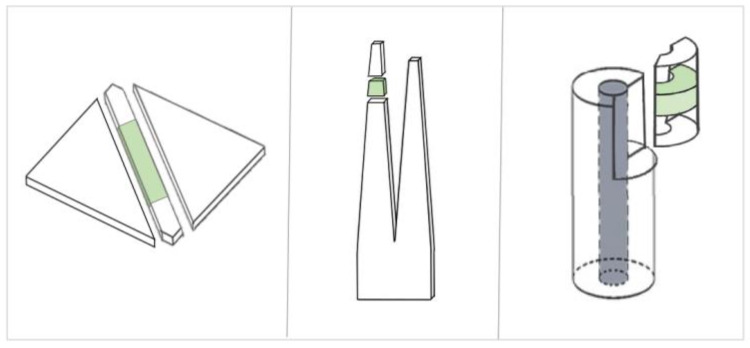
Sampling positions for the IC and GPC. The greenish areas represent the sampling position for each mock-up geometry.

Celluloid samples were taken as displayed in Figure 2. IC and GPC were performed in two measuring campaigns between September 2021 and May 2022. Four different samples were measured for each mock-up geometry before and after aging, considering at least one microsample of four mock-ups for each geometry for covering a statistically representative set.

#### 2.5.5. Ion Chromatography (IC)

Following the sample preparation proposed by Mazurek et al. [23], 5 mg of celluloid sample was dissolved in 1 mL acetone (Acetone ≥ 99.5% for synthesis, Roth) at 50 °C for one hour. The naturally aged samples (from objects P and G in Section 2.6.) did not dissolve in pure acetone; therefore, these samples were dissolved in 1 mL 7/3 (acetone:water) mixture at ca. 50 °C for two hours. The process of alkaline hydrolyses was conducted by adding 2 mL 1 N NaOH (Merck) to the previously dissolved samples and heated for 2 h at 60 °C. Finally, 17 mL water was added for a final volume of 20 mL. Before analysis, the sample solution was 100-fold diluted (0.5 mL sample solution added to 50 mL water) and then measured by a Metrohm 940 Professional IC Vario ion chromatograph (Metrohm AG, Herisau, Switzerland) equipped with a set of Metrosep columns (pre-column Metrosep A Supp 5 Guard/4.0 and main column Metrosep A Supp 7-250/4.0). The columns were maintained at 45 °C and the eluent was 3.6 mM sodium carbonate (Merck, Burlington, MA, USA) at a flow rate of 0.7 mL/min. Nitrite standard solution (Centipur^®^, Merck, Burlington, MA, USA) and the Anion multielement standard I and II (Centipur^®^, Merck, Burlington, MA, USA) were used as calibration standards.

The theory/calculation summarized by Mazurek et al. [23] was used to calculate the nitrogen content. In contrast to [23], no previous plasticizer extraction was made in this research. Therefore, for each 5 mg celluloid sample, the respective amount of camphor measured by GPC was subtracted.

#### 2.5.6. Gel Permeation Chromatography for Determining Molecular Weight (M_w_)

Based on the method of Kavda et al. [24], 2 mg of sample was dissolved in 1 mL of N,N-dimethylacetamide (DMAc 99.5% HPLC grade, Alfa Aesar) with 0.5% *w*/*v* lithium chloride (LiCl ACS 99%, Alfa Aesar) and left to dissolve overnight. Before injection, samples were filtered through a poly(tetrafluoroethylene) filter (PTFE, pore size 0.2 μm, diameter 25 mm) to remove any insoluble material and impurities. Then, 100 μL of sample solution was manually injected into a SECcurity2-GPC system (PSS). This system was equipped with a set of PSS GRAM columns (precolumn 10 μm 8 × 50 mm, 1 column 10 μm 30 Å 8 × 300 mm, and 2 columns 10 μm 1000 Å 8 × 300 mm) all sustained at 60 °C. The eluent DMAc/LiCl was maintained at a flow rate of 1 mL/min with an isocratic pump. An RID detector was set at 40 °C. The calibration with PSS ReadyCal-kit poly(methyl methacrylate) and PSS WinGPC UniChrom^®^ Software 8.3SR2 was used for the Mw estimation.

#### 2.5.7. Gel Permeation Chromatography for Determining Camphor Content

Of the sample, 5 mg was dissolved in 1 mL of tetrahydrofuran (dried and fresh distilled from technical THF) and left to dissolve for 24 h in a shaker. Before injection, samples were filtered through a poly(tetrafluoroethylene) filter (PTFE, pore size 1.0 μm, diameter 25 mm, VWR) to remove any insoluble material and impurities. Then, 20 μL of sample solution was injected by an autosampler (SECurity2 standard autosampler (404-2104), PSS) into a SECcurity2-GPC system (PSS). This system was equipped with a set of PSS SDV columns (precolumn 3 μm 8 × 50 mm, 1 column 3 μm 10,000 Å 8 × 300 mm, and 2 columns 3 μm 1000 Å 8 × 300 mm) all maintained at 25 °C. The eluent THF was maintained at a flow rate of 1 mL/min with an isocratic pump. An RID detector was set at 25 °C. The calibration with pure Camphor (Sigma Aldrich, 99+%) was done by a 5-point concentration calibration.

### 2.6. Naturally Aged Transparent Objects

**Figure 3:**
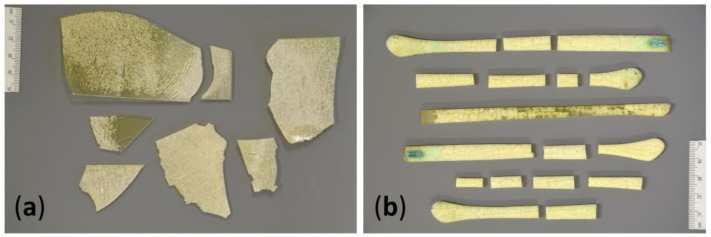
Naturally aged objects: (**a**) plates; (**b**) and temples of eyeglasses.

In this study, two collections of transparent naturally aged celluloid were considered (Figure 3), which were also already part of previous investigations by the authors Kavda et al. [24] and Elsässer et al. [16]. These materials were in extremely poor condition and fragmented, allowing extensive sampling for research purposes.

Plates (P). Transparent fragments from plates of unknown date from the former company Hutmacher and Schlund (Switzerland). In 2007, C. Heiner found these transparent plates in sealed boxes in the company’s storage area and acquired them for his private collection. In 2017, he donated several plates to the Conservation Science Department of the Deutsches Museum. The donated transparent plates have turned yellow, embrittled throughout, became severely cracked, crazed, and fallen apart in many pieces.

Temples of eyeglasses (G). Fragments of transparent glasses. The production date of the glasses is unknown, but their form and shape resemble designs of the 1950s/1960s. Until their deaccession, they belonged to the Deutsches Museum optics collection. These temples were cracked, crazed, yellowed, and presented a greenish corrosion of metal elements. Samples for analysis were taken in locations where no greenish corrosion products were visible to avoid the influence of those corrosion salts in the degradation stage of the samples and make them more comparable with the plates (P) that lacked metal elements.

## 3. Results and Discussion

### 3.1. Shaping Tines and Cylinders

**Figure 4:**
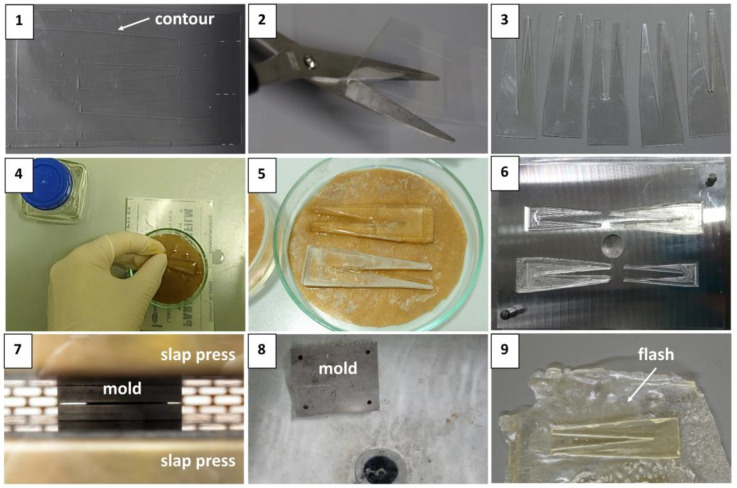
The sequence of steps for producing tines.

**Figure 5:**
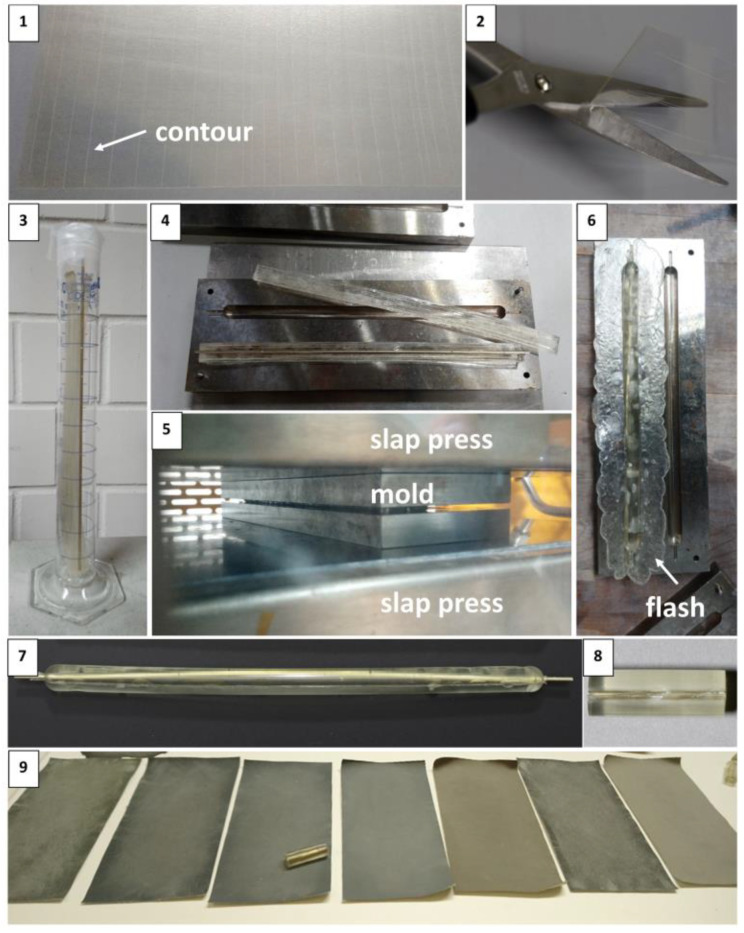
The sequence of steps for producing cylinders.

Softening trials made with granulates proved ineffective because acetone was lost too fast, reducing the working time to fill the mold and leading to undesirable defects. In contrast, the layering of several celluloid sheets led to the processing of satisfactory mock-ups. Figure 4 and Figure 5 illustrate nine steps required for making tines and cylinders. Table 5 reports the different conditions tested during the preparation of tines and cylinders.

Tines

Steps 1, 2, and 3. The contour was carved on the prefabricated celluloid sheets selected as raw materials, then cut with scissors.

Step 4. Five precut geometries were layered over each other, exceeding the desired thickness by ca. 20 vol.% to ensure complete filling of the mold after pressing. Acetone was added between layers in a ratio of approximately 1:1 (1 g celluloid to 1 mL acetone) to soften the celluloid precut geometries.

Step 5. The softened geometries were placed in petri dishes and covered with Parafilm^®^ to slow the evaporation of the solvent while being transported from the laboratory to the workshop (time between softening and pressing).

Steps 6 and 7. At the workshop, the metal mold was preheated to the set temperature in the slap press. When the desired temperature was reached, the mold was removed, and the softened geometries were inserted into the preheated mold. Then, the mold was re-replaced into the slap press. The mold stayed slightly open during preheating without pressure to allow almost complete solvent evaporation. Finally, the mold was closed for a certain pressing time under the pressure of 25 bar.

Step 8. Three main methods of cooling were available (Table 5). Step 8 in Figure 4 displays the cooling in water at approximately 20 °C.

Step 9. After cooling, the mold was opened, and the mock-ups were removed. In the final step, the excess material around the mock-ups (also called flash [50]) was removed by hand or with scissors, and the surface was polished to create a texture comparable to historical objects [26].

Cylinders

Steps 1 and 2. As for the tines, the contour was carved on the prefabricated celluloid sheets selected as raw materials, then cut with scissors.

Step 3. The geometries were layered over each other, forming the two halves of the cylinder diameter, as displayed in Figure 1. Following the same strategy as for the tines, acetone was added between layers to soften the celluloid in a ratio of approximately 1:1. After softening, a metal rod was inserted between the two halves. The softened cylinders with the metal rod were then placed in a glass tube and covered with Parafilm^®^ to slow the evaporation of the solvent while being transported from the laboratory to the workshop (time between softening and pressing).

Steps 4 and 5. At the workshop, the strategies used to preheat the metal mold to the set temperature in the slap press, fill the mold, and insert it in the slap press (preheating time, pressing time, and pressure) were the same as for the tines (steps 6 and 7).

Steps 6, 7 and 8. Three methods of cooling, as for the tines, were tested. After removal from the mold, the flash marks around the mock-ups were detached, and shorter cylinders of 3 cm in length were cut with a saw.

Step 9. The surface of the cylinders was polished with different grades of Micro-Mesh^®^ sanding sheets to create a texture comparable to historical objects [26].

Based on the visual assessment of the produced mock-ups, the optimal working parameters for compression molding were selected (Table 5). The best conditions for producing tines and cylinders were achieved by using cut sheets, softening them for one day, preheating metal mold in the slap press for 5 (tines) or 15 (cylinders) minutes, and pressing at 25 bar for 8 min at 90 °C (Table 5).

In sum, 100 tines and 100 cylinders were processed, and 70% were successfully made with nearly no visible defects (Table S1). From those, 40 mock-ups per geometry were later artificially aged. The remnant unaged 30% mock-ups with high and intermediate defects were rejected. Details of the defects are presented as Supplementary Materials (Table S2).

### 3.2. Assessing Whether the Processing Methods Induced Molecular Changes in Tines and Cylinders

The unaged tines and cylinders were investigated by ATR-FTIR to understand whether the processing could have induced unwanted heterogeneity. The values for the sheets depicted in Table 6 give the maximum allowable SD values of the absorbance intensities from the instrumental and sampling errors resulting from experiments A–C.

On one hand, all SD values attributed to the nitro groups (1634, 1273, 826 cm^−1^) were lower than the maximum allowable SD value, suggesting that no variation was detected by ATR-FTIR due to the processing of the tines and cylinders (Table 6). In addition, no significant differences were observed in the mock-ups considering the sampling locations, as the values from the top to the base for the tines or middle to edges for the cylinders were comparable.

On the other hand, the SD values of the carbonyl band (1730 cm^−1^) in the cylinders exceeded the maximum allowable limit. Steward [36] already recognized in his experiments that the carbonyl peak attributed to camphor generally shows poorer precision than the nitrate peaks. He also stated that one reason for the carbonyl band’s high variability could be the volatile nature of camphor. As such, the handling of the samples (e.g., variation in the duration of sampling and pressure during measurement on the crystal) very likely played a significant role. From these signs, the carbonyl peak could not be considered an adequate probe band to assess the possible influences due to the processing of the mock-ups.

### 3.3. Artificial Aging of Mock-Ups

This study used thermohygrometric artificial aging to accelerate deterioration processes in the newly prepared mock-ups. The cumulative effects of the aging were visually monitored to achieve a moderate deterioration stage of the mock-ups. Supposing that the mock-ups react differently to the artificial aging depending on their shape because of the different surface area to volume ratio and processing method, their results are presented separately.

**Figure 6:**
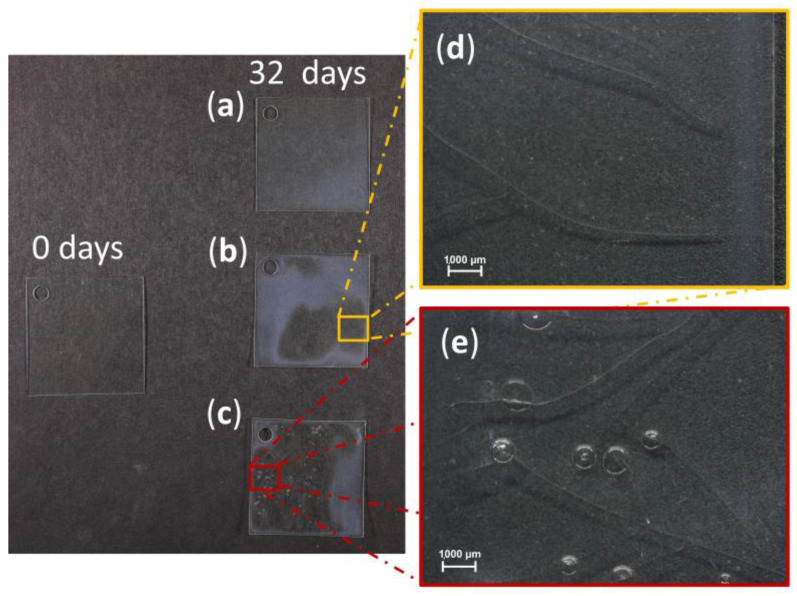
Sheets before and after 32 days of exposure to 70 °C and 75% RH. Damage phenomena observed: (**a**) loss of transparency; (**b**,**d**) loss of transparency, deformations and cracking; (**c**,**e**) loss of transparency, deformations, cracking and bubbling.

Sheets. After 32 days of exposure, different degradation phenomena were observed (Figure S2): 20 just lost their transparency (Figure 6a), 10 sheets were additionally cracked and deformed (Figure 6b,d and Figure S2), while 50 showed severe signs of degradation (Figure 6c,e and Figure S2), including loss of transparency, cracking, deformations, and bubble formation. The formation of bubbles is not a characteristic degradation phenomenon for celluloid, whereas cracks are typical of severe damage [19,20,21,29,44,51]. Aiming at mock-ups in moderate conditions with no cracks and bubbles, the 20 sheets exhibiting loss of transparency were further tested in this investigation.

**Figure 7:**
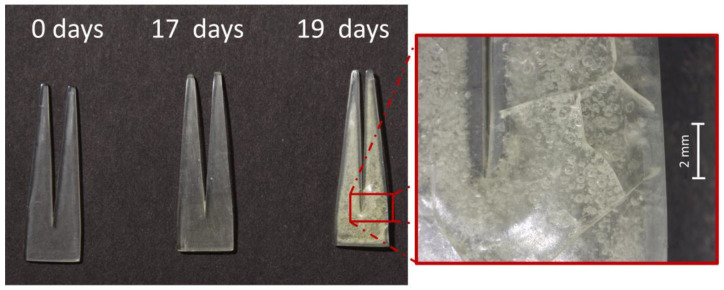
Tines before and after 17 and 19 days of exposure to 60 °C and 75% RH. Damage phenomena observed: yellowing (17 days); yellowing, bubbling, deformations and cracking (19 days).

Tines. After 17 days, all mock-ups appeared slightly yellowed, and incipient bubbles appeared in only one sample of the 40 aged ones. Only two days after, under the same conditions of artificial aging, mock-ups appeared heavily deformed, cracked, expanded and bubbled (Figure 7). Because bubbles are an unrealistic degradation phenomena, only the mock-ups aged for 17 days were considered for further analyses.

**Figure 8:**
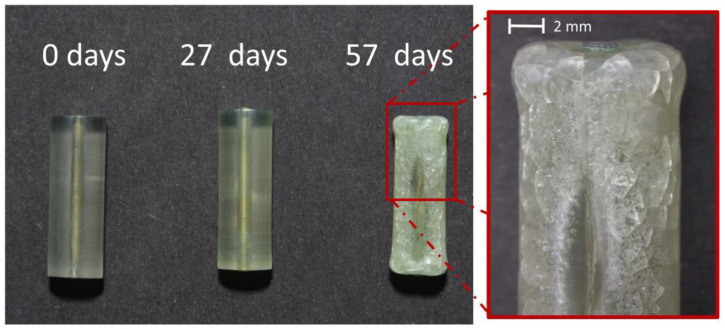
Cylinders before and after 27 and 57 days of exposure to 60 °C and 75% RH. Damage phenomena observed: yellowing and shrinkage (27 days); yellowing, bubbling, deformation, cracking, and green corrosion of the metal element (57 days).

Cylinders. The mock-ups presented the first signs of degradation after 27 days, such as yellowing and shrinkage (Figure 8). The first bubbling appeared only in one of the 40 mock-ups. A severely degraded stage was reached after 57 days in which some mock-ups appeared cracked, highly deformed, shrunk, and bubbled. Evident corrosion of the metal core was also visible. Following the same rationale as for the sheets and tines, bubbled and cracked samples were discarded, and only the 27-day-aged mock-ups considered to be in moderate condition were further investigated.

The tested environmental conditions caused bubbling in all mock-up geometries (sheets, tines, cylinders) after longer aging times, which is an unexpected degradation phenomenon. Even though bubbling is not common in celluloid degradation, the occurrence of cracks in the inner sample, is a typical macroscopic alteration of severely naturally aged objects [52]. A preliminary aging experiment yielded intense bubbling after 8 days for tines and 11 days for cylinders at 70 °C and 75% RH (more information for tines is available in Figure S1 and related paragraph). Therefore, the temperature selected to age the thicker mock-ups (tines and cylinders) was lowered to 60 °C. Nonetheless, unusual bubbles were observed at T = 60 °C (for tines and cylinders) and 70 °C (for sheets) at longer aging times. In a previous study by the authors [52], the industrial manufacturing by Rothko and Frost™ seemed to have strongly influenced the formation of bubbles and extreme volume expansion at the center of the samples made of 1 mm-thickness prefabricated sheets (made of two 0.5 mm blended sheets) due to aging at 70 °C. The 0.5 mm prefabricated sheets used in this study were not industrially layered by the company and the role played by the industrial manufacturing can be excluded. In addition, the processing method of layering used in this investigation (see Section 2.3. and Section 3.1.) did not induce volume expansion on the aged samples. Altogether, the combination of high temperatures and long aging times seemed to be the primary cause of bubbling on the mock-ups derived from the 0.5 mm prefabricated sheets, rather than the properties of the material (chemical/physical properties and formulation) or its industrial manufacturing.

To conclude, artificial aging at shorter aging times induced characteristic degradation phenomena of moderate conditions, such as loss of transparency, yellowing and slight deformation, making those mock-ups selectable for further analyses in this study.

### 3.4. Characterization of Mock-Ups after Artificial Aging

#### 3.4.1. Mass Loss

An evident mass loss after artificial aging was detected (Table 7). The mass loss at different aging times and their trend are available in the Supplementary Materials (Table S3 and Figure S4).

It is known that while celluloid degrades, it emits degradation products. In the initial phase, oxides of nitrogen (NO_x_) form [12,14,16,23,48,49,53,54,55], camphor sublimates [16,21,22,39,56], and gaseous decay products resulting from the cellulose matrix evolve [14,16,39]. The observed mass loss was consistent with the acid and camphor smell emission.

#### 3.4.2. Off-Gassing Controlling with A-D Strips

To verify that the mass loss was also due to acidic emissions (Table 7), A-D strips were closely located to the aged mock-ups in closed bags. After 24 h, they turned yellow, confirming that the mock-ups were off-gassing. Their condition state not only fits the requirements of a moderate state of degradation for this investigation but is also common in historic celluloid collections [43,44].

#### 3.4.3. Assessment of Degradation via ATR-FTIR

**Figure 9:**
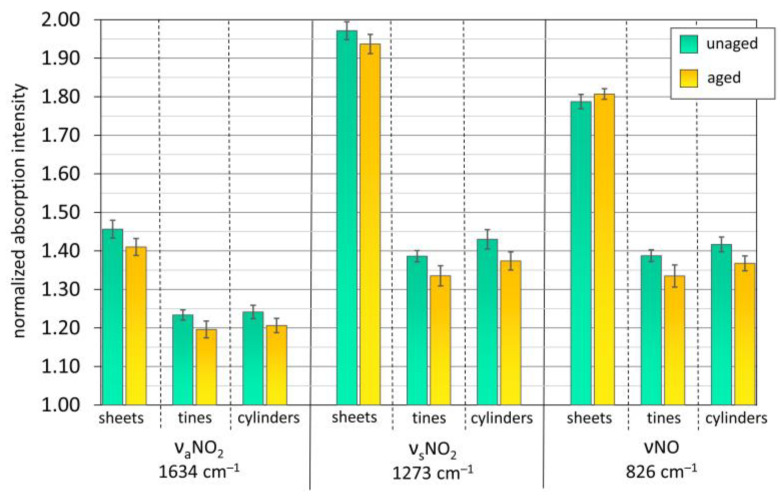
Normalized ATR-FTIR absorbance intensity for unaged and aged mock-ups concerning the nitrate probe bands. The error bars correspond to the SD. Aging conditions: sheets 70 °C, 75% RH, 32 days; tines 60 °C, 75% RH, 17 days; cylinders 60 °C, 75% RH, 27 days.

Figure 9 and Figure S4 show a decrease in the nitrate vibrations probe bands’ intensities, except for the νNO 826 cm^−1^ solely for the sheets. The nitrate vibration probe bands for all other mock-ups showed a consistent but slight decrease along aging, which corroborates the assumption that incipient-induced degradation occurred and was likely due to the homolytic scission of the nitro groups (denitration) [12,48,49]. As presented by [52], the monitoring of the nitrate bands in ATR-FTIR can be used as a semiquantitative strategy to assess celluloid degradation, particularly when unaged and severely degraded samples are compared. In this study, ATR-FTIR showed limitations in estimating the degradation extent of the artificially aged mock-ups because of their moderate condition.

**Figure 10:**
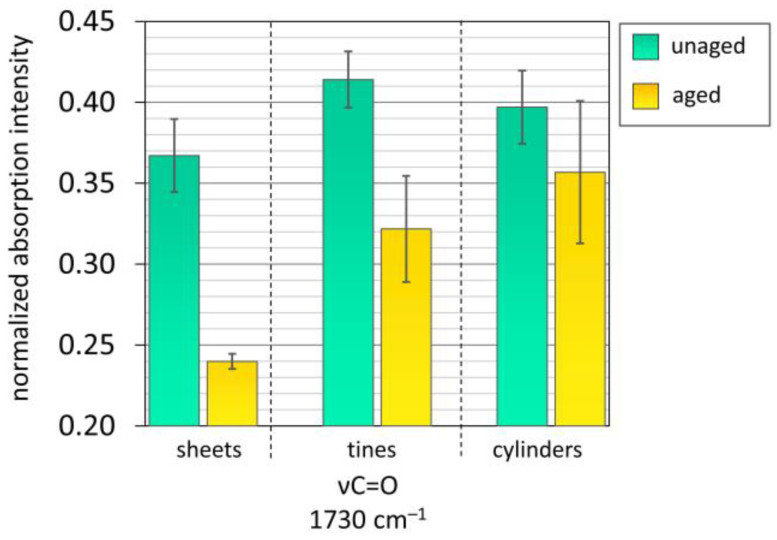
Normalized absorbance intensity for unaged and aged mock-ups concerning the carbonyl probe band. The error bars correspond to the SD. Aging conditions: sheets 70 °C, 75% RH, 32 days; tines 60 °C, 75% RH, 17 days; cylinders 60 °C, 75% RH, 27 days.

The carbonyl peak at 1730 cm^−1^ showed an evident decrease after aging (e.g., more than 34% for the sheets; Figure 10). This decrease might be partly attributed to the loss of the plasticizer camphor due to sublimation. However, the intensity of the carbonyl peak is not solely affected by the concentration of plasticizer but also by the extent of degradation of the polymer chain. Indeed, as cellulose nitrate degrades, carbonyl groups are formed at the cellulose nitrate chain contributing to absorbance at 1730 cm^−1^ [33,48,52,54]. The intensity of this band can cause a misleading interpretation regarding the loss of plasticizer. A recent publication by the authors about the aging of similar prefabricated celluloid samples by ATR-FTIR analysis showed an apparent increase in the C=O contribution due to severe deterioration [52]. Therefore, GPC was used for quantifying camphor in this study.

#### 3.4.4. Characterization of the M_w_, Nitrogen, and Camphor Contents through Chromatographic Methods

Table 8 summarizes the results obtained by IC and GPC. All unaged mock-ups are characterized by higher M_w_, camphor, and nitrogen contents than the artificial aged ones. These results indicate that homolytic scission of nitro groups (denitration), reduction of the cellulose nitrate M_w_ (*β*-glucoside chain cleavage), and loss of the camphor took place.

In general, the M_w_ displayed the most significant differences between unaged and aged mock-ups, with a decline of 59.0% (sheets), 19.8% (tines), and 33.5% (cylinders) compared to the unaged values. Even though it is commonly agreed in the literature that the initial step of CN degradation involves the homolytic scission of the O-NO_2_ due to its low bonding energy [57,58], as depicted by Neves et al. [48], the reduced M_w_ values suggest that the mock-ups in moderate condition were subjected to intense *β*-glucoside chain cleavage. The predominance of the chain cleavage compared to denitration in celluloid degradation has already been observed [23,59].

#### 3.4.5. Comparison of the Mock-Ups with 3D Naturally Aged Objects

**Figure 11:**
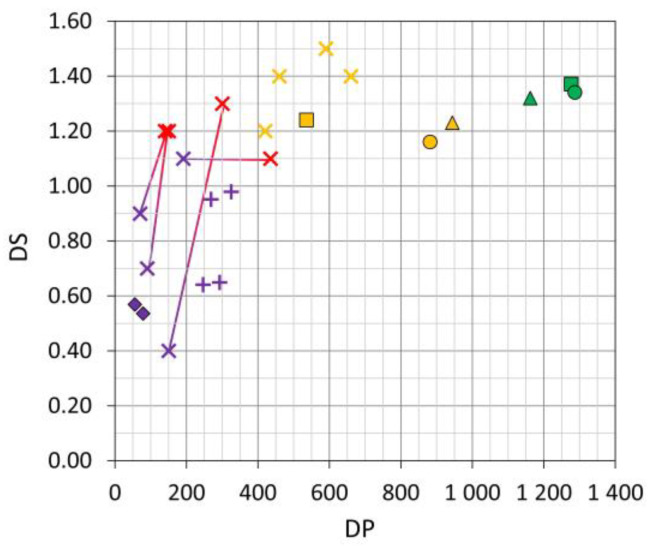
Degree of substitution (DS) and degree of polymerization (DP) of the mock-ups (□ sheets, ∆ tines, o cylinders) and 3D naturally aged celluloid objects: from the DM collection (◊), from [23] (x) and from [22] (+). The condition state of the mock-ups and samples from [22,23] were classified in green (good, no off-gassing); yellow (good, off-gassing); red (severe, areas physically not damaged); and purple (severe, areas physically damaged), adapted from [23,44]. Purple and red symbols are connected with a solid line when they derive from the same object.

Nitrogen contents (used for calculating the degree of substitution-DS), M_w_ (employed for determining the degree of polymerization-DP), and camphor contents of 3D naturally aged objects made of celluloid are seldom reported in the literature. In contrast, extensive literature data are available for 2D cinematographic films and photographs, which is hardly comparable with the aging behavior of 3D mock-ups. In this study, the DS and DP of the artificially aged mock-ups were compared with data of 3D naturally aged celluloid objects: (a) published in [22,23] and (b) measured in eyeglasses and plates from the DM. The DS and DP were calculated according to [23] and plotted in Figure 11. Based on [23,44], four main categories of condition state were adapted and used in this study for the mock-ups and the 3D naturally aged objects:

- green color code: visibly in good condition without acid detection
- yellow color code: visibly in good condition but with acid detection
- red/purple color codes: visibly in poor condition, exhibiting signs of degradation and acids detection. Data plotted in red and purple can derive from samples taken from the same object, displaying the heterogeneous nature of celluloid degradation. Such decay pattern has been already reported in collections [16,19,21,23,27,43,52]. Red corresponds to areas where macroscopically no physical damage appeared, while purple refers to areas that are physically damaged, cracked, or crazed.

Based on the previously described categories, the unaged mock-ups were classified as green, the artificially aged ones as yellow, and the naturally aged objects from the DM as purple. The data from [22], described in that article as being collected from very or highly degraded objects (with cracks and disintegrating in several pieces), were here classified as purple.

The results indicate that the unaged mock-ups have the highest DP, directly related to a high M_w_. It is important to notice that their DP absolute values are hard to corroborate with historical sources from the first half of the 20th century, when celluloid was mainly produced, as size exclusion chromatography was only invented in 1955 and no information on the M_w_ before that time is available. The raw material, manufacturing, and processing conditions strongly influence the final M_w_ of cellulose nitrate. Considering literature from 1968, DP can considerably vary from 100 to 1300 [60].

The aged mock-ups show DP values between ca. 500 and 900, which is slightly higher than the DP values of naturally aged objects in moderate conditions (400–700) considering the classification by [23,44]. In comparison, the historical objects classified as being in poor condition are characterized by the lowest DP values.

Considering the DS values, it is hard to recognize a clear trend between moderately (1.5–1.2) and severely aged (1.3–0.4) artifacts. However, Figure 11 clearly emphasizes the heterogeneous nature of severely degraded celluloid within the same object.

Even though the M_w_ and DS values of the historical objects after production and their extent of decrease due to aging are unknown, a trend in the reduction of both DP and DS can be suggested along the aging, which is underlined by the data plotted from the severely naturally aged objects ([22,23] and DM collection). If the DP and DS values of all those historical objects in purple and red are representative of highly degraded historical objects, then one can infer that the artificially aged mock-ups are in a moderate condition because the values are ranked between unaged and severely aged celluloid (Figure 11).

Camphor contents in celluloid have mainly been mainly addressed by IR spectroscopy [13,22,61], which is a not straightforward strategy due to concurrent influences on the carbonyl band vibration. In fact, camphor loss and formation of carbonyl-containing degradation products [52] can both at the same time affect the intensity of this vibration due to aging.

**Figure 12:**
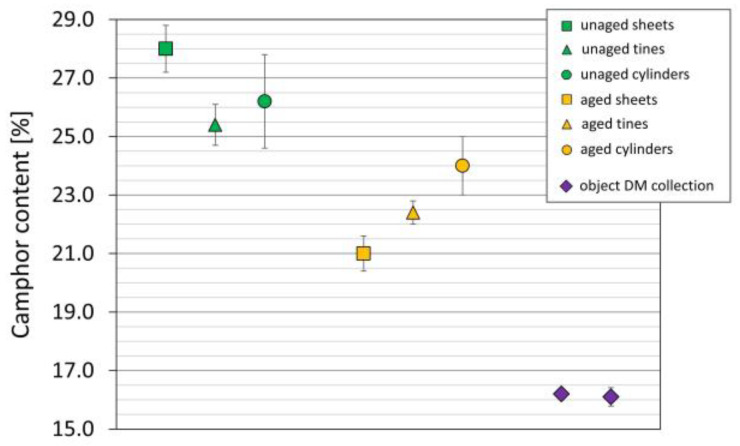
Camphor content (%) of the mock-ups and naturally aged glasses and plates.

In this study, the camphor content of artificially aged mock-ups and naturally aged objects calculated by a quantitative method (GPC) are compared. The camphor contents of the sheets, tines, and cylinders decreased due to aging 25%, 12%, and 8%, respectively (Figure 12). The aged sheets displayed the most significant loss. Ca. 16% of camphor was detected in the naturally aged objects from the DM. Bearing in mind that the highest camphor value for 3D objects is around 33% [16,21,62,63], the artificially aged mock-ups would resemble moderately aged objects.

## 4. Conclusions

This work emphasizes the need and advantages of processing mock-ups in celluloid conservation research with shapes resembling 3D historical artifacts. The 3D celluloid mock-ups, namely, sheets, tines, and cylinders, referring to swatches, combs, and eyeglass temples, were successfully made by combining historical industrial processing strategies and small-scale lab facilities. Different compression molding variables and combinations were tested, which were crucial for determining the best processing conditions, leading to 70% satisfactory results. After processing, tines and cylinders were analyzed by ATR-FTIR, and the nitro group probe bands showed no detectable variation due to shaping, suggesting that the developed protocol (layering, softening, compression molding and cooling) enabled their reproducible creation. The processing protocol developed in this study can be considered adequate for creating celluloid mock-ups in plastic heritage studies, to investigate not only their aging behavior but also to explore the effectiveness of passive and active conservation treatments.

The artificial aging experiments were adequate for inducing aged mock-ups (being yellowed and off-gassing) capable of simulating moderately degraded historical objects, as verified by comparing published and original data from chemical analyses of 3D objects from collections. Besides detecting acidic emissions, IC and GPC confirmed denitration, loss of camphor, and polymer chain scissions. The chain cleavage was predominant compared to denitration. The moderately aged mock-ups are currently being investigated within a research project [15], which is dedicated to systematically assess the effectiveness of cool, cold, and frozen storage conditions for celluloid 3D objects without testing unique and valuable historical artifacts.

This work paves the way for the use of 3D celluloid mock-ups in heritage science studies and can inspire their future application in research on celluloid. In this regard, mock-ups with different material compositions (e.g., different plasticizer concentrations and filler contents) and geometries would support aging and conservation studies. In addition, it opens new avenues for investigating unresolved research questions about the complex decay processes of 3D celluloid. A heterogeneous nature at advanced stages of alteration has been observed for 3D celluloid objects, leading to degradation gradients recognizable by the naked eye [12,15,17,19,35,44,52]. Even though this decay phenomenon is considered typical, it has been studied very little until now. Considering mock-ups as reliable testing materials, research on severely degraded 3D celluloid mock-ups would support a better understanding of the nature of this decay. To this end, mock-ups can be systematically aged under different environmental conditions to simulate historical objects in poor conditions. In doing so, it is advisable to consider the glass-transition temperature (Tg) of celluloid in selecting the temperature of the artificial aging protocol to avoid unwanted degradation phenomena (e.g., bubbling). The chemical characterization of mock-ups in comparison with naturally aged historical objects through spectroscopic and chromatographic methods is essential for verifying their ability to simulate the state of 3D celluloid in collections. The availability of mock-ups simulating different degradation stages will serve to explore conservation treatments, e.g., cleaning, consolidation, planning of proper showcases in exhibitions and tailored storage solutions, towards a better preservation of complex 3D celluloid artifacts for future generations.

## Figures and Tables

**Table 1:** Dimensions of the mock-ups.

Sheets	Tines	Cylinders Celluloid Composite with Metal Core
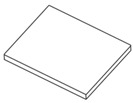	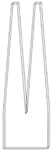	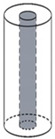
40 (L) × 40 (W) × 0.5 (D) mm	50 (L) × 16 (W) × 2 (D) mm	Height: 30 mm; Ø = 10 mm

**Table 2:** Applied temperatures and relative humidity used in thermohygrometric aging of cellulose esters in heritage science studies.

Reference	Temperature [°C]	Relative Humidity [%]	Type *	Exposure Time
Shashoua et al. 1992, [33]	60, 70, 100	n/a	CN	Up to 80 days
Hamrang 1994, [34]	50, 60, 70, 80	0, 15, 30, 40, 50, 100	CN	Up to 60 weeks
Derrick 1994, [35]	50	55	CN	75 days
Steward et al. 1996, [36]	70	75	CN	n/a
Steward 1997, [37]	70	12, 55, 75	CN	Up to 14 days
Quye et al. 2011, [12]	70	12, 55, 75	CN	Up to 14 days
Richardsson et al. 2013, [38]	70	33, 54, 70	CA	Up to 66 days
Curran et al. 2018, [39]	80	65	CN, CA	Up to 10 weeks
Kemper and Lichtblau 2019, [40]	70	50	CA	Up to 82 days
Da Ros et al. 2021, [41]	70	50, 80	CA	Up to 120 days

* CN is a common acronym in cultural heritage science studies and often refers to either cellulose nitrate or celluloid. CA means cellulose acetate.

**Table 3:** Measuring spots on unaged celluloid sheets to determine the SD values of instrumental (experiment A) and sampling (experiments B and C) errors by ATR-FTIR.

Experiment A	Experiment B	Experiment C
10 x same spot	10 positions on the same sheet	One position on 10 sheets
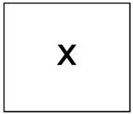	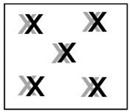	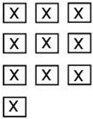

Note: X measuring spot on one side of the sheets; X measuring spot on the other side of the sheets.

**Table 4:** Measuring and sampling strategy implemented for the ATR-FTIR investigation of the mock-ups after production, before and after artificial aging.

Mock-Up Geometry	Sheet	Tine	Cylinder
Spectra acquisition	Direct measurement on the mock-up	Measurement of a sampled chip	Measurement of a sampled chip
	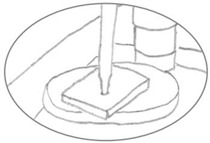	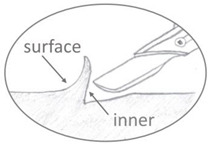	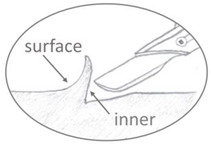
Location of the measuring and sampling points	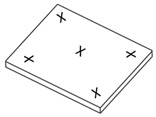	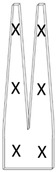	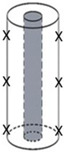

**Table 5:** Working parameters tested for producing tines and cylinders. The optimal working parameters are highlighted in bold.

Parameters	Tested Settings	Evaluation of Results
Raw material	Granulates	insufficient connection, crowning
	Sheets	best condition
Time between softening and pressing	4 h	too much acetone gas, more bubbles, and cavities
	1 day	best condition
	2 days	starting to harden, material tensions
	6 days	hardened, strong material tensions
Set temperature of the slap press	90 °C	best condition
	100 °C	more bubbles and cavities
	120 °C	many bubbles, material tensions, deformations
Preheating time	3 * and 10 ** min	too much acetone remaining
	5 * and 15 ** min	best condition
	7 * and 20 ** min	not enough acetone remaining, time-consuming
Pressure	0 bar	insufficient connection
	25 bar	best condition
Pressing time	5 min.	insufficient connection, more bubbles, and cavities
	8 min	best condition
	10 min	harder to demold, time-consuming
Method of cooling	No–directly open in the air	harder to demold, time-consuming
	Slow-Slab press	time-consuming
	Fast-Water quench	best condition

* Tines. ** Cylinders.

**Table 6:** Normalized absorbance intensities of the ATR-FTIR probe bands of sheets, tines, and cylinders. The sheets, neither manipulated by the shaping process nor the aging, set the maximum allowable SD value for normalized absorbance intensities for each ATR-FTIR probe band calculated from experiments A–C.

	1730 cm^−1^ νC=O	1634 cm^−1^ ν_a_NO_2_	1273 cm^−1^ ν_s_NO_2_	826 cm^−1^ νNO
	Avg. ± SD	Avg. ± SD	Avg. ± SD	Avg. ± SD
sheets	0.367 ± 0.020	1.442 ± 0.024	1.951 ± 0.034	1.739 ± 0.025
tines	0.414 ± 0.017	1.234 ± 0.013	1.386 ± 0.015	1.388 ± 0.015
cylinders	0.397 ± 0.023	1.241 ± 0.017	1.430 ± 0.025	1.417 ± 0.019

Note: avg. average; SD standard deviation; ν stretching; ν_a_ antisymmetric stretching; ν_s_ symmetric stretching.

**Table 7:** Mass loss [%] after artificial aging.

Mock-Up Geometry	Sheets	Tines	Cylinders
Aging conditions	70 °C, 75% RH	60 °C, 75% RH	60 °C, 75% RH
Exposure time	32 d	17 d	27 d
Mass loss	–9.4% ± 1.5	–2.6% ± 0.23	–3.5% ± 0.13

**Table 8:** Results of the chromatographic analyses of the unaged and aged mock-ups.

	Aging	M_w_ (GPC)		Camphor Content (GPC)		Nitrogen Content (IC)	
		Avg. ± SD [Da]	RSD	Avg. ± SD [%]	RSD	Avg. ± SD [%]	RSD
sheets	unaged	283,000 ± 16,900 (n = 6)	6.0%	28.0 ± 0.4 (n = 4)	1.3%	8.66 ± 0.13 (n = 4)	1.5%
	aged *	116,000 ± 10,500 (n = 6)	9.1%	21.0 ± 0.6 (n = 6)	2.9%	7.80 ± 0.17 (n = 4)	2.2%
tines	unaged	257,000 ± 9,400 (n = 6)	3.6%	25.4 ± 0.7 (n = 4)	2.8%	8.23 ± 0.13 (n = 4)	1.6%
	aged *	206,000 ± 24,900 (n = 6)	12.1%	22.4 ± 0.4 (n = 6)	2.0%	7.82 ± 0.16 (n = 4)	2.1%
cylinders	unaged	284,000 ± 15,300 (n = 6)	5.4%	26.2 ± 1.6 (n = 4)	6.0%	8.28 ± 0.14 (n = 4)	1.7%
	aged *	189,000 ± 26,800 (n = 6)	12.2%	24.0 ± 1.0 (n = 6)	4.1%	7.71 ± 0.16 (n = 4)	2.1%

Note: avg., average; SD, standard deviation; n, number of measurements; RSD, relative standard deviation. *Aging conditions: sheets 70 °C, 75% RH, 32 d; tines 60 °C, 75% RH, 17 d; cylinders 60 °C, 75% RH, 27 d.

## Data Availability

Data available on request.

## Supplementary Materials

The following supporting information can be downloaded at https://www.mdpi.com/article/10.3390/polym15040852/s1, Figure S1: Preliminary trial of artificial aging of tines at 70 °C and 75 % RH after 6, 7 and 8 days. Bubbling was clearly visible after 7 days and increased excessively after 8 days; Figure S2: Deformations of the sheets after 32 days of exposure to 70 °C and 75 % RH. Deformations were observed at sheets showing: (a) loss of transparency and cracking and (b) loss of transparency, cracking and bubbling; Figure S3: Diagram addressing the time-dependent mass loss of mock-ups; Figure S4: Averaged infrared spectra before and after artificial ageing of (a) sheets (n=20) (b) tines (n=24) and (c) cylinders (n=24). Arrows indicate a decrease in the probe bands after artificial ageing; Table S1: Overview of the rejected and selected mock-ups based on their production defects; Table S2: Production defects that appeared during the preparation of mock-ups; Table S3: Calculated mass loss in % during artificial ageing of the mock-ups shaped as sheets. Tines and cylinders.
