# Supplementary material for: Mock-Ups in Plastic Conservation Research: Processing and Aging of 3D Celluloid Specimens Simulating Historical Objects
Source: Polymers (Basel). 2023 Feb 8;15(4):852. doi: 10.3390/polym15040852 (PMC9960551; doi:10.3390/polym15040852)
Supplement: Supplementary file 1 [file polymers-15-00852-s001.zip › polymers-2120053-supplementary.pdf]

Supplementary information

Mock-ups in plastic conservation research: processing and aging of 3D celluloid specimens simulating historical objects

Christina Elsässer [1,2], Veronika Mayr [1], Peter Montag [3], Eva Mariasole Angelin [1], Harald Hilbig [4], Christian Ulrich Grosse [2] and Marisa Pamplona [1,*]

1 Conservation Science Department, Deutsches Museum, Museumsinsel 1, 80538 Munich, Germany
2 Chair of Non-destructive Testing, Technical University of Munich, Franz-Langinger-Straße 10, 81245 Munich, Germany
3 PSS, Polymer Standards Service GmbH, In der Dalheimer Wiese 5, 55120 Mainz, Germany
4 Professorship of Mineral Construction Materials, Technical University of Munich, Franz-Langinger-Straße 10, 81245 Munich, Germany;
* Correspondence: m.pamplona@deutsches-museum.de

Artificial aging of tines - preliminary trial

	6 days	7 days	8 days
**Tines**	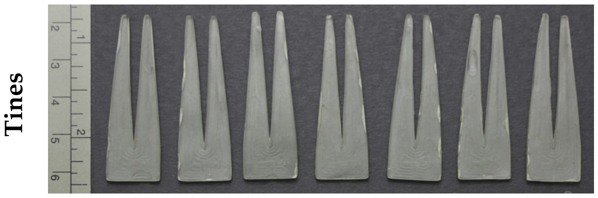	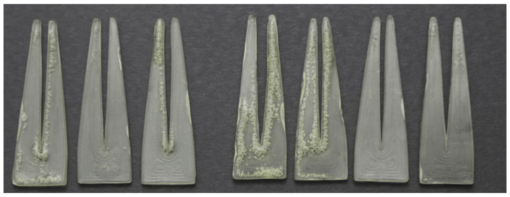	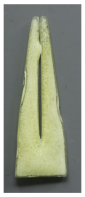

Figure S1. Preliminary trial of artificial aging of tines at 70 °C and 75 % RH after 6, 7 and 8 days. Bubbling was clearly visible after 7 days and increased excessively after 8 days.

Bubbling occurred likely because the glass transition temperature (Tg) was exceeded. One can infer that the Tg was lowered by the conditions applied during the artificial aging. The high relative humidity might have led to an increased distance between polymeric chains due to absorbed water. Therefore, the mobility of the polymer chains was increased, and their Tg was lowered. In addition, it was unclear how the artificial aging influenced the mock-ups' camphor content and their chemical and mechanical properties over time. Indeed, a decrease in the molecular weight and nitro groups would increase the mobility of the polymer chains. This would result in lower interchain attraction and cohesion due to the enlarged free volume of the end group regions.

Table S1. Overview of the rejected and selected mock-ups based on their production defects.

rating	defects	examples
Selected mock-ups (no deformation) ca. 70 %	• No bubbles, blowholes, deformation or discoloration<br>• Only small cavities on sites and surface of tines<br>• Only small sunken areas on cylinders	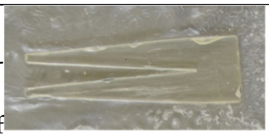 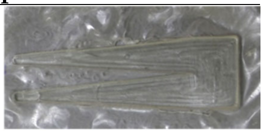 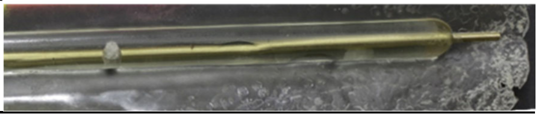

Rejected mock-ups (intermediate deformation) ca. 10 %	• Very few small bubbles<br>• Small blowholes<br>• Small sunken areas on cylinders<br>• Irregularities at the surface<br>• Little deformation	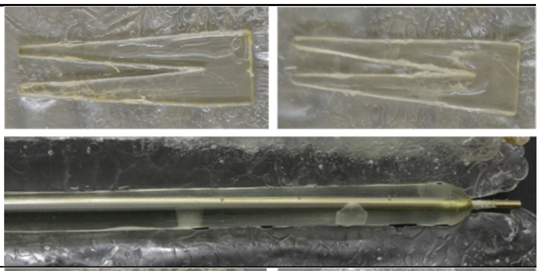
Rejected mock-ups (high deformation) ca. 20 %	• Large bubbles and/or many small bubbles<br>• Large blowholes<br>• Sunken areas on cylinders<br>• Strongly decentred metal rods<br>• Strong deformation<br>• discoloration	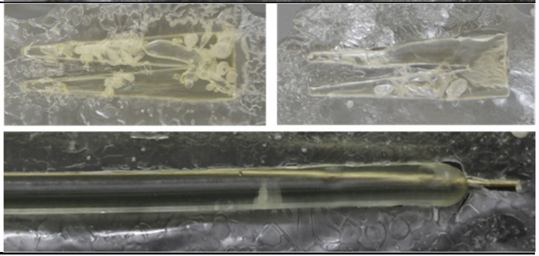

Table S2. Production defects that appeared during the preparation of mock-ups.

Technical Term	Definition and cause	Examplery mock-up
**Cavities**	Air trapped in the mold during filling, cannot escape in time an accumulates between surface and mold; insufficient ventilation of the mold	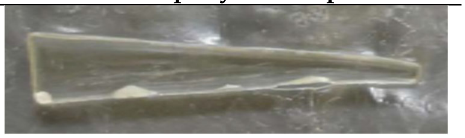
**Incomplete filling**	Geometry not fully formed; insufficient injection pressure in the case of injection molding, insufficient (over)filling in the case of compression molding	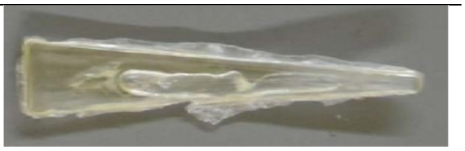
**Blowholes**	Individual or closely spaced cavity-like fissured vacuum spaces, create sink marks below the surface; in injection molding due to insufficient holding pressure, in compression molding due to insufficient pressure	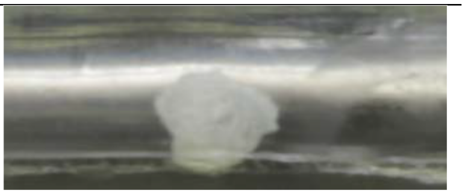
**Bubbles**	Formed by thermally or chemically induced material decomposition (here by accumulation of acetone or press temperature of 120 °C or above)	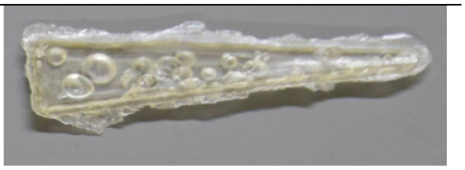
**Deformation due to demolding**	Deviation from dimensional accuracy; due to the application of too much force during demolding, incorrect tool geometry or too soft molding	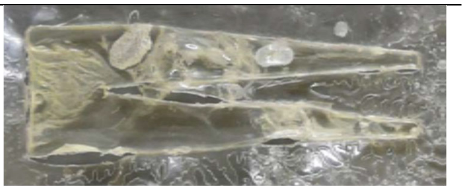

**Shrinkage/ warp-age**	Deviation from dimensional accuracy; due to (local) shrinkage due to inhomogeneous material thickness, temperature or pressure gradients, regression of molecular orientations or too high press temperature; shortening of all dimensions during cooling	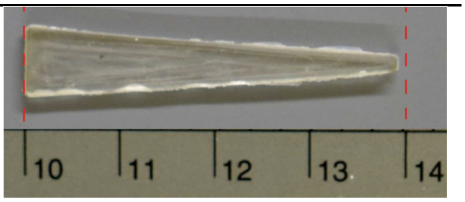
**Crown**	Inhomogeneities in the volume; due to unmelted granulate, here hardly distinguishable from blow-holes	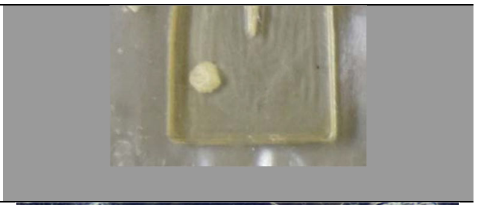
**Material tensions**	Molecular orientations in the material; here due to the effect of pressure on hardened preform, tension is "frozen" in the material during cooling (image: mock-up between crossed polarized filters)	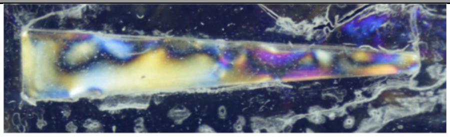
**Formation of fringes**	Compacted area near the surface, thickness usually up to 0.1 mm; due to cold mold inner wall or irregular cooling from outside to inside, here caused by water bath	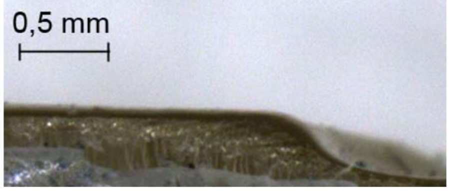
**Soiling with rust**	Rust particles on the surface or in the volume; due to non-rust-resistant tool and insufficient cleaning	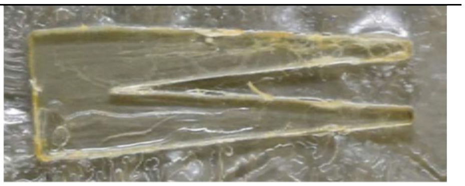

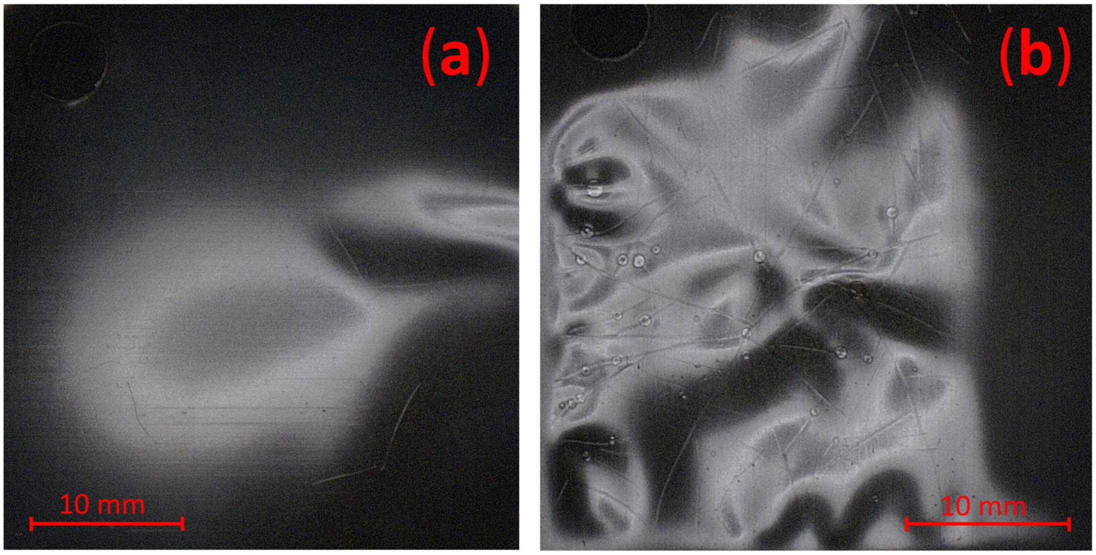

Figure S2. Deformations of the sheets after 32 days of exposure to 70 °C and 75 % RH. Deformations were observed at sheets showing: (**a**) loss of transparency and cracking and (**b**) loss of transparency, cracking and bubbling.

Table S3. Calculated mass loss in % during artificial ageing of the mock-ups shaped as sheets. Tines and cylinders.

	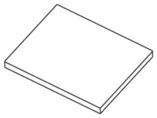 **(n=8)**			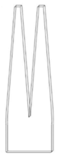 **(n=8)**			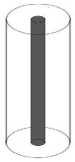 (n=8)		
**Days of exposure**	**Ave.**	**St.Dev.**	**RSD**	**Ave.**	**St. Dev.**	**RSD**	**Ave.**	**St. Dev.**	**RSD**
**1**	NM	NM	NM	-0,1 %	± 0,1	62,6 %	0,2 %	± 0,0	9,6 %
**2**	NM	NM	NM	-0,7 %	± 0,1	13,9 %	0,0 %	± 0,0	85,8 %
**3**	-1,3 %	± 0,3	19,7 %	-1,1 %	± 0,1	9,5 %	-0,2 %	± 0,0	24,6 %
**4**	-1,6 %	± 0,3	17,1 %	NM	NM	NM	NM	NM	NM
**5**	-1,9 %	± 0,3	15,9 %	NM	NM	NM	NM	NM	NM
**6**	-2,2 %	± 0,3	14,3 %	-1,8 %	± 0,1	6,0 %	-0,8 %	± 0,1	8,6 %
**7**	-2,4 %	± 0,3	13,5 %	-1,9 %	± 0,1	6,1 %	-0,9 %	± 0,1	7,7 %
**8**	NM	NM	NM	-2,0 %	± 0,1	6,2 %	-1,0 %	± 0,1	7,2 %
**9**	NM	NM	NM	-2,2 %	± 0,1	5,8 %	-1,1 %	± 0,1	6,8 %
**10**	-2,9 %	± 0,3	11,7 %	-2,3 %	± 0,1	5,1 %	-1,3 %	± 0,1	6,2 %
**11**	-3,2 %	± 0,3	11,0 %	NM	NM	NM	NM	NM	NM
**12**	-3,3 %	± 0,4	10,7 %	NM	NM	NM	NM	NM	NM
**13**	-3,4 %	± 0,4	10,3 %	-2,5 %	± 0,1	4,1 %	-1,5 %	± 0,1	5,3 %
**14**	-3,5 %	± 0,4	10,0 %	-2,5 %	± 0,1	4,6 %	-1,6 %	± 0,1	5,1 %
**15**	NM	NM	NM	-2,5 %	± 0,1	4,3 %	-1,7 %	± 0,1	5,0 %
**16**	NM	NM	NM	-2,5 %	± 0,1	5,2 %	-1,8 %	± 0,1	4,8 %
**17**	-3,9 %	± 0,4	9,0 %	-2,6 %	± 0,2	9,1 %	-1,9 %	± 0,1	4,9 %
**18**	-4,2 %	± 0,4	8,6 %				-2,0 %	± 0,1	4,8 %
**19**	-4,3 %	± 0,4	8,1 %				-2,0 %	± 0,1	5,1 %
**20**	-4,5 %	± 0,3	7,7 %				-2,0 %	± 0,1	5,4 %
**21**	-4,7 %	± 0,4	7,6 %				-2,1 %	± 0,1	5,2 %
**22**	NM	NM	NM				-2,3 %	± 0,1	4,4 %
**23**	NM	NM	NM				-2,3 %	± 0,1	4,4 %
**24**	-5,1 %	± 0,4	6,8 %				-2,4 %	± 0,1	4,0 %
**25**	-5,4 %	± 0,4	6,7 %		*No further ageing*		-2,5 %	± 0,1	3,7 %
**26**	-5,6 %	± 0,4	6,3 %				-2,3 %	± 0,1	4,4 %
**27**	-5,9 %	± 0,4	6,2 %				-2,3 %	± 0,1	4,4 %
**28**	NM	NM	NM						
**29**	NM	NM	NM						
**30**	NM	NM	NM					*No further ageing*	
**31**	NM	NM	NM						
**32**	-9,4 %	± 1,5	15,8 %						

NM not measured.

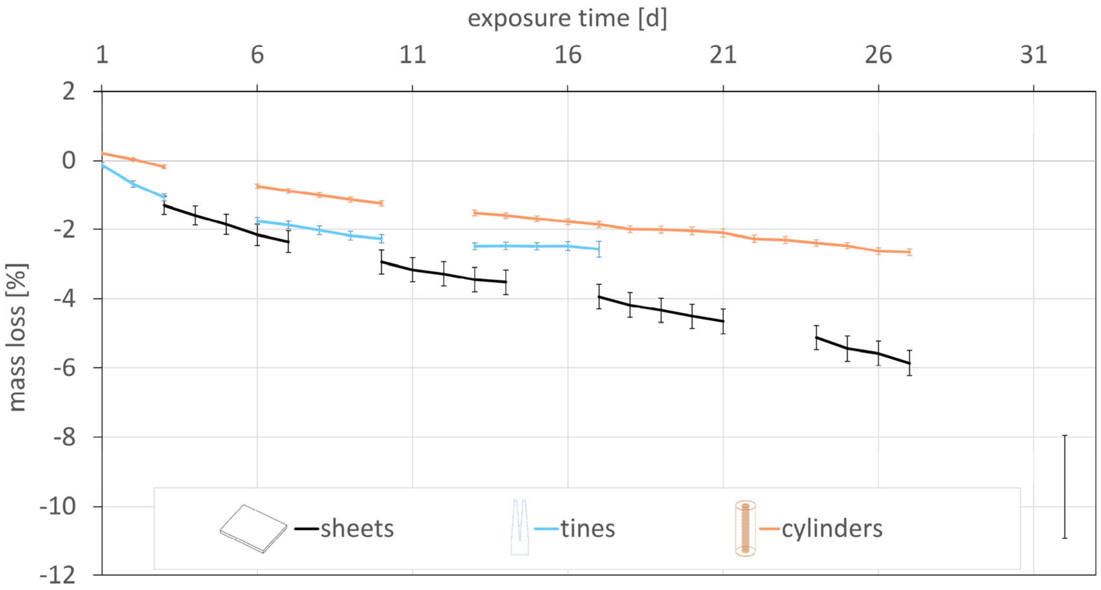

Figure S3. diagram addressing the time-dependent mass loss of mock-ups.

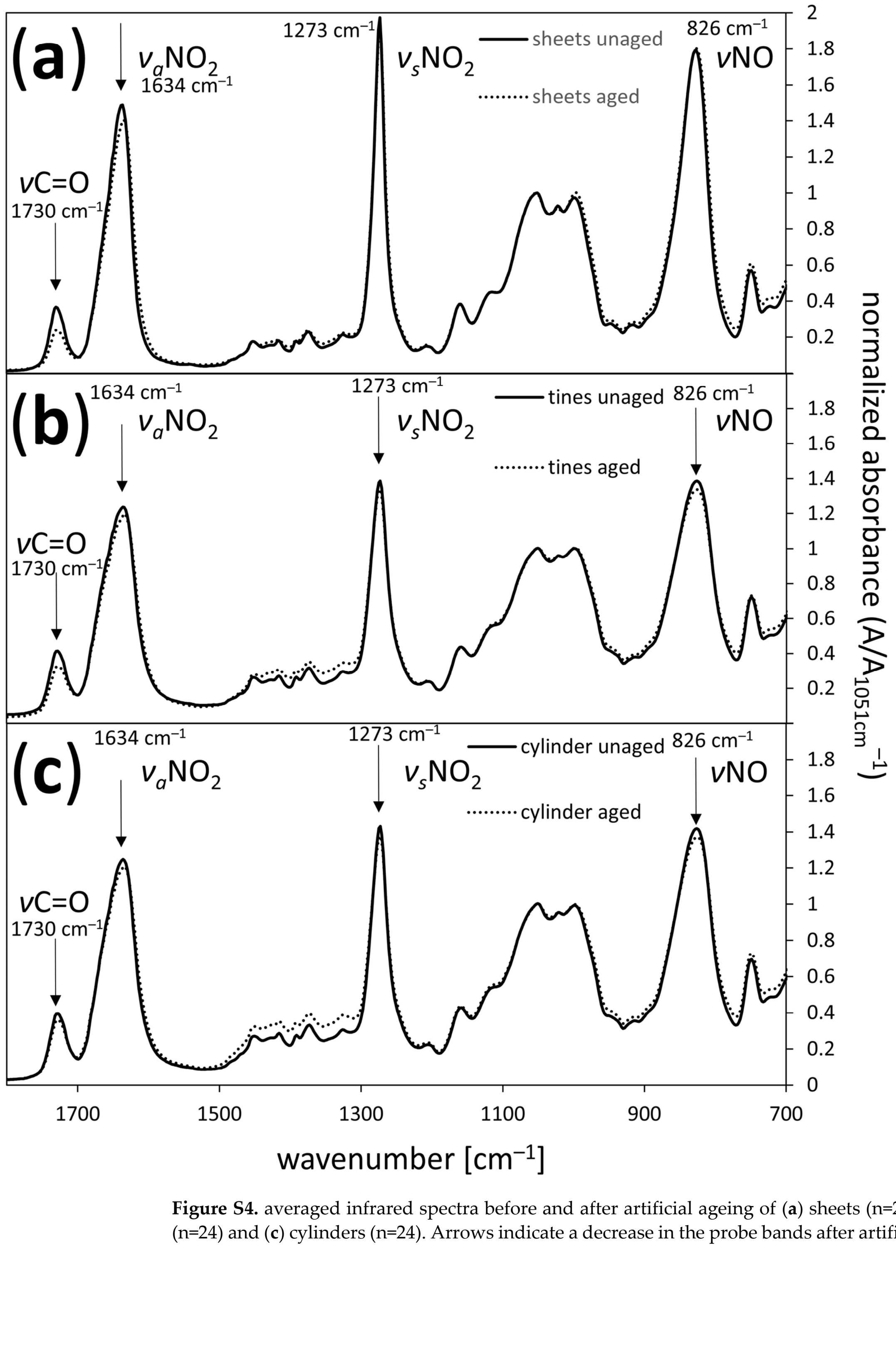

Figure S4. averaged infrared spectra before and after artificial ageing of (**a**) sheets (n=20) (**b**) tines (n=24) and (**c**) cylinders (n=24). Arrows indicate a decrease in the probe bands after artificial ageing.
